# Reframing obstetric practice: a call to action for the paradigm of great obstetrical syndromes

**DOI:** 10.1097/j.pbj.0000000000000304

**Published:** 2025-10-20

**Authors:** Renato Augusto Moreira de Sa, Edward Araujo Junior

**Affiliations:** a Department of Clinical Research, Instituto Nacional de Saúde da Mulher, da Criança e do Adolescente Fernandes Figueira, Rio de Janeiro (RJ), Brazil; b Department of Obstetrics and Gynecology, Unidade Local de Saúde Santa Maria, Lisboa, Portugal; c Department of Obstetrics, Universidade Federal de São Paulo, Escola Paulista de Medicina, São Paulo (SP), Brazil

Obstetric practice is at crossroads. The long-dominant symptom-based model of managing pregnancy complications must now give way to a mechanism-oriented approach, centered on the concept of the Great Obstetrical Syndromes (GOS)^1^. First introduced over a decade ago, GOS represent a mechanism-based framework encompassing conditions such as preeclampsia, fetal growth restriction (FGR), preterm labor, placental abruption, stillbirth, recurrent miscarriage, and gestational diabetes mellitus (GDM). Though clinically distinct, these syndromes share fundamental features: multifactorial origins, early subclinical onset, failed physiological adaptations, and—in most cases—placental dysfunction^1,2^.

Important mechanisms related to GOS include inflammation and defective deep placentation, especially inadequate remodeling of spiral arteries, which leads to oxidative stress and an anti-angiogenic state. Elevated soluble fms-like tyrosine kinase-1 (sFlt-1) and reduced placental growth factor (PlGF) levels create maternal endothelial dysfunction, impair fetal development, and drive systemic effects—most clearly in preeclampsia and FGR^2,3^. Even in GDM, traditionally considered metabolic, placental pathology reveals vascular involvement. Histologic findings such as villous immaturity, chorangiosis, and ischemia point to altered placental structure and function^4^. These abnormalities compromise nutrient exchange, potentially contributing to macrosomia and long-term metabolic programming in the offspring^5^.

A key advantage of the GOS framework is its focus on early prediction. First-trimester screening, incorporating maternal risk factors, uterine artery Doppler, and biochemical markers, like PlGF and pregnancy-associated plasma protein-A, identify high-risk pregnancies before clinical signs appear^6^. Low-dose aspirin, initiated between 11 and 14 weeks in high-risk women, has demonstrated efficacy in reducing preterm preeclampsia—an example of how early identification can enable prevention^6^. In GDM, lifestyle and nutritional interventions, even if not fully preventive, reduce associated complications such as macrosomia^4^.

The introduction of angiogenic biomarkers, particularly the sFlt-1/PlGF ratio, has refined diagnostic and prognostic accuracy in obstetrics. In suspected preeclampsia, this ratio helps confirm diagnosis and guide delivery timing^3,6^. In FGR, elevated sFlt-1/PlGF levels support differentiation between true placental insufficiency and constitutionally small fetuses^2^. Even in cases of preterm labor and preterm prelabor rupture of membranes, abnormal angiogenic profiles may indicate underlying placental disease in the absence of infection^3^.

Although available only postnatally, placental histopathology offers retrospective validation of clinical suspicions and improves biomarker interpretation^3^. In GDM, placental changes may persist despite good glycemic control, suggesting that glucose alone does not reflect the full disease burden^4^. Integrating placental findings into obstetric practice elevates the understanding of syndromic origins and supports more informed counseling for future pregnancies.

The most compelling reason to adopt the GOS framework is its relevance beyond birth. Women with GOS—especially preeclampsia or GDM—face increased lifetime risk of cardiovascular disease, metabolic syndrome, and type 2 diabetes. Their children also carry elevated risks of obesity, hypertension, and chronic disease, reinforcing the Developmental Origins of Health and Disease hypothesis^5,6^.

It is time to embrace a practical and clinically useful approach to Great Obstetrical Syndromes, grounded in mechanistic understanding while respecting the distinct characteristics of each condition revealed by advances in precision medicine. To meet the challenge of GOS, obstetricians must integrate early screening using history, Doppler, and biomarkers; apply preventive interventions such as aspirin and lifestyle guidance; use biomarkers judiciously, guided by placental phenotype; value the placenta as a dynamic organ central to disease; and foster continuity of care, ensuring follow-up for both mother and child.

The placenta is not merely a passive conduit but an active participant in health and disease. Recognizing and acting upon the framework of the Great Obstetrical Syndromes can improve outcomes for generations.
